# Neurophysiology tools to lower the stroke onset to treatment time during the golden hour: microwaves, bioelectrical impedance and near infrared spectroscopy

**DOI:** 10.1080/07853890.2022.2124448

**Published:** 2022-09-25

**Authors:** Lazzaro di Biase, Adriano Bonura, Maria Letizia Caminiti, Pasquale Maria Pecoraro, Vincenzo Di Lazzaro

**Affiliations:** a Department of Medicine and Surgery, Unit of Neurology, Neurophysiology and Neurobiology, Università Campus Bio-Medico di Roma, Roma, Italy; b Fondazione Policlinico Universitario Campus Bio-Medico, Roma, Italy; c Brain Innovations Laboratory, Università Campus Bio-Medico di Roma, Rome, Italy

**Keywords:** Stroke, golden hour, stroke onset to start of treatment time, near infrared spectroscopy (NIRS), bioelectrical impedance spectroscopy, volumetric impedance phase shift spectroscopy (VIPS), microwave imaging (MWI)

## Abstract

Reperfusion therapy administration timing in acute ischaemic stroke is the main determinant of patients’ mortality and long-term disability. Indeed, the first hour from the stroke onset is defined the “golden hour”, in which the treatment has the highest efficacy and lowest side effects. Delayed ambulance transport, inappropriate triage and difficulty in accessing CT scans lead to delayed onset to treatment time (OTT) in clinical practice. To date brain CT scan is needed to rule out intracranial haemorrhage, which is a major contraindication to thrombolytic therapy. The availability, dimension and portability make CT suitable mainly for intrahospital use, determining further delays in the therapies administration. This review aims at evaluating portable neurophysiology technologies developed with the scope of speeding up the diagnostic phase of acute stroke and, therefore, the initiation of intravenous thrombolysis. Medline databases were explored for studies concerning near infrared spectroscopy (NIRS), bioelectrical impedance spectroscopy (BIS) and Microwave imaging (MWI) as methods for stroke diagnosis. A total of 1368 articles were found, and 12 of these fit with our criteria and were included in the review. For each technology, the following parameters were evaluated: diagnostic accuracy, ability to differentiate ischaemic and haemorrhagic stroke, diagnosis time from stroke onset, portability and technology readiness level (TRL). All the described methods seem to be able to identify acute stroke even though the number of studies is very limited. Low cost and portability make them potentially usable during ambulance transport, possibly leading to a reduction of stroke OTT along with the related huge benefits in terms of patients outcome and health care costs. In addition, unlike standard imaging techniques, neurophysiological techniques could allow continuous monitoring of patients for timely intrahospital stroke diagnosis.

**KEY MESSAGES**
- First hour from the stroke onset is defined the “golden hour”, in which the treatment has the highest efficacy and lowest side effects.
- The delay for stroke onset to brain imaging time is one of the major reasons why only a minority of patients with acute ischaemic stroke are eligible to reperfusion therapies.
- Neurophysiology techniques (NIRS, BIS and MWI) could have a potential high impact in reducing the time to treatment in stroke patients.

## 1. Introduction

Stroke is the second leading cause of death and the major cause of disability in the world, with an annual mortality of 5.5 million [1]. In 2017, the stroke-related costs was 45.5 billion USD in USA [2] and 60 billion Eur in Europe, with health care accounting for 27 billion EUR (45%), representing 1.7% of health expenditure [3].

**Figure 1.**
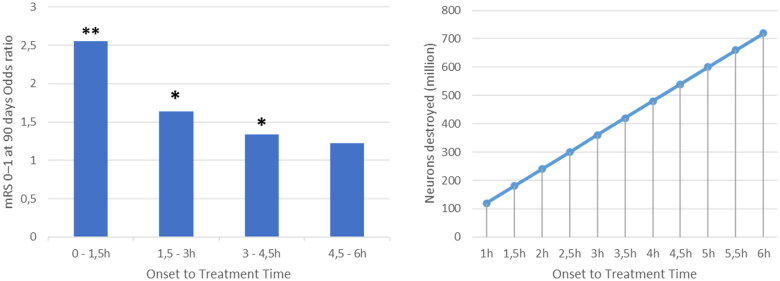
The Graph on the left and the one on the right, have been created by the authors of the present article in order to synthesize in a graphical way the result of the data reported in [5] (left graph) and [6] (right graph). Graph on the left: time to treatment with intravenous alteplase and outcome in stroke patients. Pooled analysis of ECASS, ATLANTIS, NINDS and EPITHET trials [5]. Data adjusted for stroke onset to start of treatment, National Institutes of Health Stroke Scale score at baseline, age, diastolic blood pressure, previous hypertension, previous stroke and interaction of age and NIHSS score. The odds ratio of modified Rankin (mRS) score (0–1) at 90 d were 2,55 for 0–90 min, 1,64 for 91–180 min, 1,34 for 181–270 min and 1,22 for 271–360 min in favour of the alteplase group [5]. Legend: **p<.001; *p<.05. Graph on the right: Neurons destroyed (million) in relation to stroke onset to revascularization treatment time [6].

The majority (87%) of stroke are ischaemic [2]. Despite ischaemic stroke is a potentially treatable disease, thanks to available treatments, the mortality and disability related to this disease are still very high worldwide. The applicability and therapeutic efficacy of revascularization therapies, such as thrombolysis and thrombectomy, which represent the standard of care in the acute phase of ischaemic stroke, are strictly time dependent [4]. “Time is brain” summarizes very well the need for an immediate intervention in ischaemic stroke. Indeed, every single minute after stroke onset on average 12 km (7.5 miles) of myelinated fibres, 1.9 million neurons and 14 billion synapses are lost, resulting in an accelerated brain ageing of 3.6 years each hour without treatment (Figure 1) [6,7].

Every 15 min delay in the administration of reperfusion therapy after stroke onset leads to an increased risk of long-term disability, in-hospital mortality and intraparenchymal haemorrhage [8]. For these reasons, the first hour from the stroke onset is defined the “golden hour”, in which the treatment has the highest efficacy and lowest side effects [7]. Pooled analysis from ECASS [9], ATLANTIS [10], NINDS [11] and EPITHET [12] trials showed how the reduction in timing from stroke onset to start of treatment (OTT) with somministration of alteplase (rTPA) is associated with a reduction in disability at 90 d compared with placebo (Figure 1) [5]. In addition, this study showed a statistically significant (*p*=.04) association between OTT and mortality, with a significant deaths increase at OTT higher than 270 min [5]. In clinical practice, however, timely intervention is not always possible. In USA, estimates of reperfusion therapy rate in acute stroke have ranged from 3 to 5% [13,14], in some hospital can range as high as 20–30% [15,16]. Several studies have shown how factors such as inappropriate triage, the presence of fluctuating symptoms, delay in ambulance transport can delay and difficulty in accessing a CT image can increase OTT [17]. In addition, although it is widely accepted that a stroke patient needs rapid diagnostic workup in order to start the treatment as soon as possible if no contraindications are present, there is an inverse relationship between the time from symptoms onset to emergency department arrival and the time from emergency department arrival to tPA treatment [18,19]. Which means that patients who arrive at the emergency department soon after stroke onset typically have longer delays between arrival and initiation of tPA therapy [18,19].

Imaging methods, such as brain CT is fundamental in the first diagnostic phase of stroke. Although in acute onset CT scan does not always allow to document the presence of a stroke (or may only show indirect signs), it can rule out an intracranial haemorrhage that represents the main contraindication to the revascularization therapies [4,20,21]. To discern clinically between ischaemic and haemorrhagic stroke is often not possible, so it makes necessary to have a test with high diagnostic accuracy in recognizing and differentiating the two pathologies. The high sensitivity and specificity of brain CT and MRI in the differential diagnosis between ischaemic and haemorrhagic stroke are counterbalanced by their availability, dimension and portability that make them suitable mainly for intrahospital use. The suggested, door to CT imaging interpretation timing, for emergency department stroke management is 45 min aiming to a door-to-treatment of 60 min [22]. However, we need to add the onset-to-door time to this time, considering that only around the 30% of stroke patients arrive in timing to emergency department for the thrombolysis time windows [23–33]. The main strategy to increase the usage and the efficacy and at the same time lower the potential side effect of revascularization therapies, is to minimize the stroke OTT.

For this reason, the development of portable new technologies for acute stroke diagnosis, and especially in the rule out of a haemorrhagic stroke, has become a hot topic with the aim of prehospital stroke diagnosis and earlier administration of thrombolytic therapy even during ambulance transfer, which could lead to a gain of about 1–2 h in OTT.

Even for large vessels occlusion (LVO) stroke requiring mechanical thrombectomy, it has been shown that administration of thrombolytic pre-thrombectomy (in the time window <4.5 h from symptom onset) is associated with better functional outcomes at 90 d than thrombectomy alone [34]. Different technologies have been proposed with the aim of speeding up the diagnostic phase of stroke. Although portable version of classic brain imaging techniques, like CT [35] and MRI [36,37] scanners are available, their still high dimension and costs limit their usage in clinical practice.

Clinical neurophysiology techniques using electromagnetic [38,39] or ultrasound waves [40,41] to study brain activity [42] currently used for research purposes have a potential diagnostic [43] and therapeutic applications [44,45], these techniques are portable and have lower cost in respect of standard brain imaging techniques.

For stroke diagnosis, the most promising neurophysiological techniques explored until now are near infrared spectroscopy (NIRS), bioelectrical impedance spectroscopy (BIS) and microwave imaging (MWI). The purpose of the present review is to evaluate the possible impact of these technologies on stroke management during the golden hour and their potential readiness level for the application in clinical practice.

## 2. Methods

### 2.1. Data source and selection

A MEDLINE (PubMed) literature review was performed to identify all published studies about NIRS, BIS and MWI.

The string described in Table 1 was used in PubMed search engine, for article selection.

**Table 1.** Articles search strategy.

Domain	Search string
Technology	(“Near Infrared Spectroscopy” OR “NIRS” OR “Ultra-wideband” OR “Ultra wide band” OR “UWB” OR “microwave imaging” OR “MWI” OR “Volumetric Impedance Phase Shift spectroscopy” OR “VIPS” OR “Bioelectrical Impedance Spectroscopy” OR “Bioelectrical Impedance” OR “Bioimpedance”) AND
Stroke diagnosis	((“stroke” OR “cerebral ischemia”) OR
Haemorrhage diagnosis	(“hemorragic” OR “hematomas” OR “hemorrhage”))

We have included articles regarding NIRS, BIS and MWI in acute ischaemic or haemorrhagic stroke diagnosis. We have also included articles regarding non-traumatic intracerebral haemorrhagic events as their presence is a main contraindication to revascularization therapies. We limited our selection to clinical humans’ studies excluding in lab simulations on brain models and animal experiments. For each of the three technologies the following data were collected: Stroke *vs.* healthy and ischaemic stroke *vs.* haemorrhagic events diagnostic performance, spatial resolution, depth of penetration, time from stroke onset resolution, technology readiness level (TRL).

**Figure 2.**
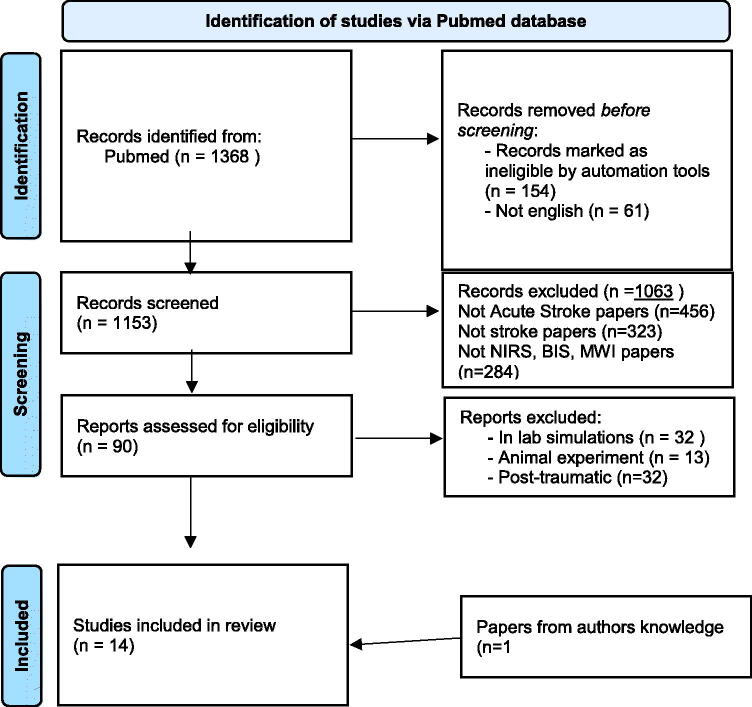
Flow diagram: results of articles search strategy.

We found 1368 total articles (Figure 2). Reviews, letters, editorial, commentaries and books or non-English papers were excluded before screening. A careful evaluation of the title and abstract was done, and studies that did not address acute stroke diagnosis were excluded. Were excluded also 32 studies regarding in lab simulation, 13 studies regarding animal experiments and 32 studies regarding post-traumatic haemorrhage or haematomas.

After evaluation of inclusion and exclusion criteria, 12 studies were included in the present review, and another one from authors knowledge: 7 about NIRS, 3 about on BIS and 3 about MWI.

## 3. Neurophysiology technologies

### 3.1. Near infrared spectroscopy (NIRS)

NIRS is a diagnostic method for biological tissues oxygenation study [46]. Electromagnetic waves, in the frequency band between 700 and 900 nm, are released by the device and cross the biological tissue generating two phenomena: scattering and absorption. Scattering depends on the wave-matter interaction that causes changes in the direction of the photons. This can lead to the reflection and the exit from the biological material of the wave that could be detected by NIRS sensors or to the absorption of the wave. The latter consists in the transfer of the wave energy to the material [47].

In biological tissues, absorption refers mainly to haemoglobin according to the absorption coefficients which depends on haemoglobin oxygenation degree. This characteristic allows to measure the two forms of Hb, therefore, the oxygen saturation in the tissue. The wavelength of 805nm is the isosbestic point of Hb, at this wavelength the absorbance value is the same for both oxygenated and deoxygenated haemoglobin. Therefore at this wavelength is possible to measure blood volume [47].

The maximum distance between the end of the detector fibre and the end of the fibre emitting the optical radiation is usually 3.5–4 cm, allowing NIR photons to penetrate the underlying biological tissue to a maximum depth of 3–3.5 cm [48].

NIRS has been mainly used in cerebral oxygenation investigation with different purposes including study of brain activity (functional NIRS as an alternative method to fMRI) [49] and detection of cerebral hypoxia in newborns [50], and diagnosis of dementia disease such as Alzheimer Disease [51] and monitoring during thrombectomy [52].

Moreover, FDA has approved the use of NIRS for detection of traumatic haematomas with a sensitivity of 78%, specificity of 90%, positive predictive value of 77% and negative predictive value of 90%. However, there are several limitations to its use: and the most relevant is the penetration depth <2.5 cm, this limitation allows NIRS to detect only cortical, epidural or subdural haematomas [53].

There are only few papers about the use of NIRS in ischaemic stroke diagnosis. Moreau et al. [54] conducted a clinical study on five patients with acute cerebral ischaemia (mean onset time: 3.5 h) and analyzed them with quantitative frequency domain NIRS (fdNIRS or Q-NIRS) method that, modulating the wave frequency, is able to quantify the percentage of tissue oxygenation. Sensors were placed in frontal, parietal and temporal bone level. Results showed reduction in oxyhaemoglobin saturation (StO2) in at least one brain area involved by ischaemic stroke (analyzed by CT) in all five patients with significantly lower absolute StO2 values than healthy controls (11 patients). One patient developed haemorrhagic transformation of ischaemic stroke and underwent hemicraniectomy. NIRS-detected values of StO2 and total tissue haemoglobin (O2Hb + HHb) significantly increased in haemorrhagic stroke patient compared with patients with ischaemic stroke and healthy controls [54].

Quantitative fdNIRS was also used to detect cerebral ischaemia in 25 patients undergoing neurovascular surgery. Five patients had ischaemic events during the procedure, and in all cases fdNIRS detected decreased perfusion in terms of increased deoxyhemoglobin and decreased oxyhaemoglobin [55]. Another study with fdNIRS showed reduced absolute StO2 values in acute stroke patients (*n* = 5) compared with controls (*n* = 11). The study also evaluated StO2 in five cadavers that showed significantly reduced values compared with controls and you have acute stroke patients [54].

Several studies have shown that NIRS can detect cerebral hypoafflux and consequently stroke in patients undergoing cardiovascular surgery. Erdoes et al. [56] used two emitter-detector units of a non-quantitative NIRS system positioned in the forehead of patients undergoing cardiopulmonary bypass or cardiac surgery affected by stroke in the postoperative period. Results show that NIRS was not able to detect eight strokes. In these patients, the ischaemic lesions were mainly localized in the posterior areas of the temporal and parietal lobes, in the occipital lobe, and in the corona radiata (as watershed stroke). Failure in recognizing the localization of specific areas involved in the ischaemic injuries was pointed to the poor spatial coverage of the sensors [56]. In another study conducted on 47 patients undergoing aortic arch surgery and monitored with QNIRS, it was shown that a drop in StO_2_ was closely related to the occurrence of neurological events [57].

**Figure 3.**
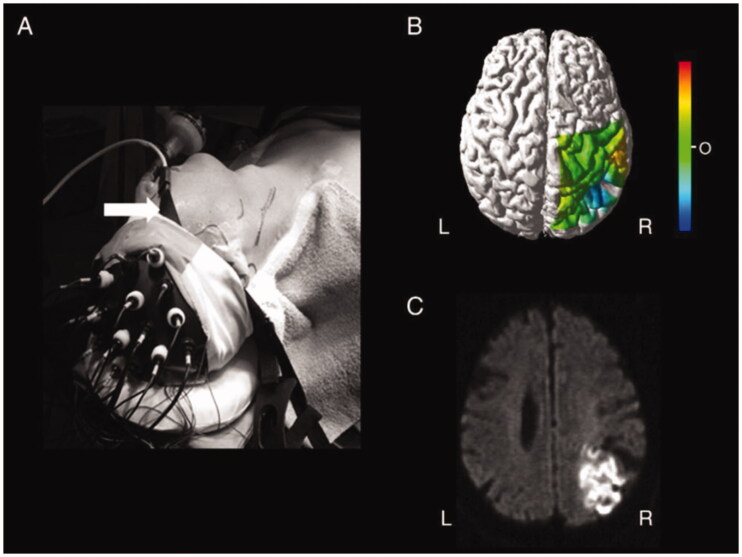
Multichannel NIRS optical topography perioperative stroke detection. (A) The placement of the optodes (OMM 2000) and the sensor (INVOS 3110 A) during surgery. White arrow indicates the sensor of the INVOS 3110 A. (B) Optical topographic maps demonstrate decreases in Oxy-Hb in the watershed zone between the MCA and PCA on the right side during cross-clamping of the ICA. The maps were overlaid on anatomical MRI surface images. C: Diffusion-weighted MRI shows high intensity regions corresponding to the area associated with the decrease of Oxy-Hb. Abbreviations: ICA, internal carotid artery; MCA, middle cerebral artery; Oxy-Hb, oxyhaemoglobin; PCA, posterior cerebral artery; OT, optical topography. [Reprinted from Nakamura et al. [59] with permission from Elsevier].

In another study conducted on 59 patients undergoing aortic surgery and monitored with a two-sensor NIRS system, there were 16 cases of stroke detected by a reduction in StO2 and 1 undetected case. The latter was caused by embolism in a brain area not covered by NIRS [58]. The use of multiple recording channels NIRS was evaluated in a single 67-year-old patient, during a carotid endarterectomy (CEA) procedure (Figure 3), multichannel NIRS optical topography was able to identify a posterior watershed stroke not detected by single channel NIRS [59].

NIRS (Infrascan Model 1000) has already been approved by the FDA for the diagnosis of superficial cerebral haematomas and is currently on market [53]. There are several NIRS models on the market but none of them is validated as a diagnostic method for ischaemic stroke. Indeed NIRS as stroke diagnosis system is at level 7 of the TRL scale since there are clinical trials and a technology transfer but no large trials have been performed that allow to validate the technology [60].

Possibility of using a contrast agent to increase the diagnostic accuracy of NIRS was also evaluated. Indocyanine green and NIRS were used in 13 patients to detect anterior circulation stroke. The difference in Time to Peak and Time interval between stroke patients and controls showed excellent sensitivity and specificity [61].

### 3.2. Bioelectric impedance spectroscopy

**Figure 4.**
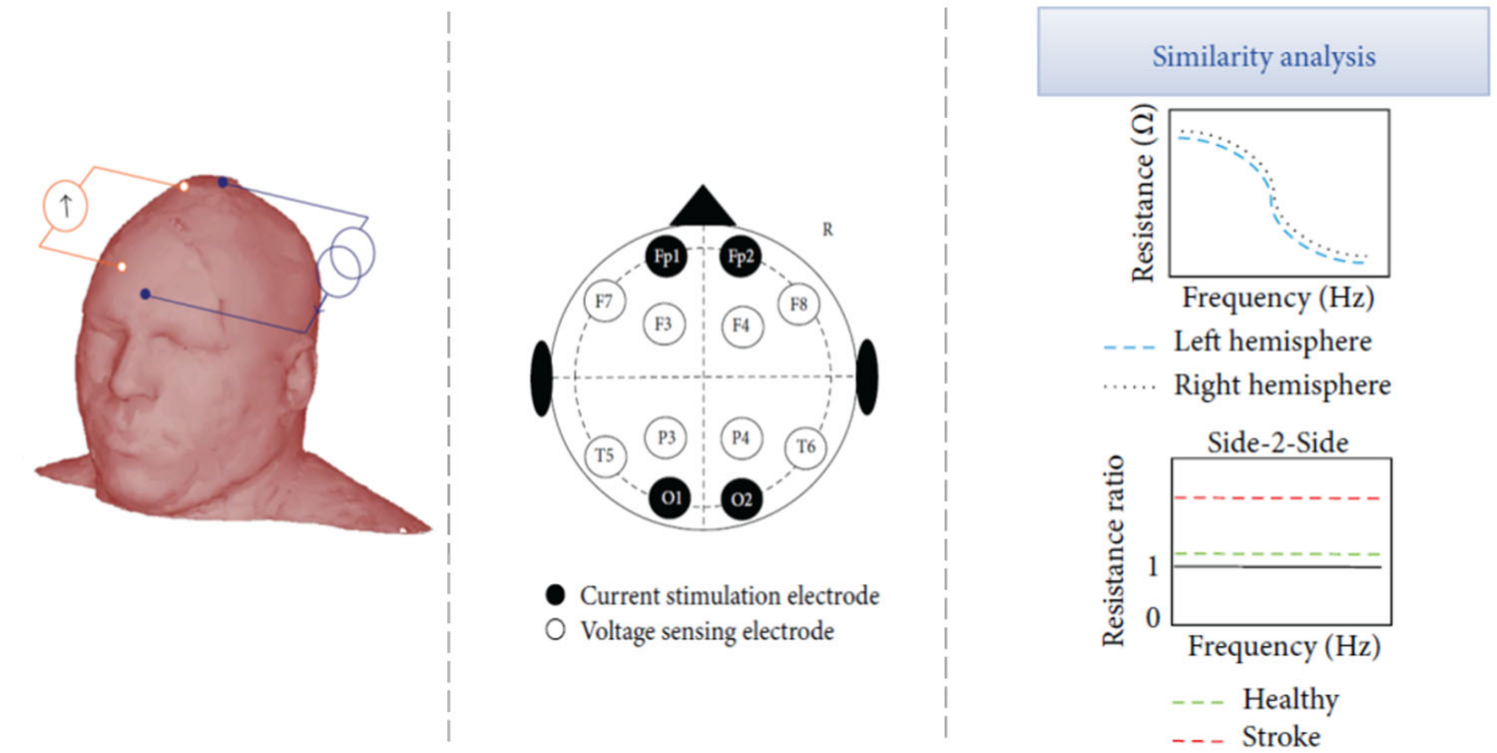
Bioelectric impedance spectroscopy. From the left the figure shows the tetrapolar method for non-invasive bioimpedance measurements. One pair of electrodes is used for electrical stimulation and another pair of electrodes senses the resulting voltage difference between the sensing electrodes. The central image shows the location of current stimulation electrodes couples (filled circles) and the voltage sensing electrodes couples (hollow circles) according to the 10–20 international EEG landmarks. The right image shows the bioimpedance analysis, which compare the resistance between the two hemispheres of two homologous tetrapolar measurements, in stroke patients the resistance ratio is higher than healthy subjects. (modified under the terms and conditions of the Creative Commons Attribution (CC BY) licence from Seoane et al. [64]).

Electrical impedance represents the oppositional force of a body to the passage of alternating current. Determination of bioimpedance is primarily used to discriminate body composition [62]. Animal studies showed differences in impedance between healthy and stroke-affected brain tissue [63]. In particular, ischaemic tissue is characterized by an increase in electrical impedance compared with healthy tissue (within 1–3 h of stroke onset), whereas haemorrhagic tissue is characterized by a reduction in electrical impedance, as early as 10 min after onset [63]. Detection of brain tissue bioimpedance was implemented by several methods. This technology compare the bioimpedance in the two hemispheres and exploiting the asymmetric nature of stroke events, can detect through an asymmetry algorithm a difference in bioimpedance related to a stroke event [64–66]. Seoane et al. [64] and Atefi et al. [65] conducted two clinical studies using the SF7B spectrometer by impedimed [67] that transmits electrical sinusoidal waves at a frequency of 3096–1000 kHz, and is a BIS-based technology without phase shift. In these studies [64,65], a tetrapolar method for non-invasive bioimpedance measurements has been used, one pair of electrodes is used for electrical stimulation and another pair of electrodes senses the resulting voltage difference between the sensing electrodes, the electrodes are placed according to the 10–20 international EEG landmarks (Figure 4). Both studies showed significant changes in ischaemic tissue impedance in stroke patients, these alterations were not present in healthy controls. Seoane et al. [64] also included haemorrhagic strokes, but it was not possible to differentiate between subjects with ischaemic stroke from those with haemorrhagic stroke [64].

Volumetric impedance phase shift spectroscopy (VIPS) technology owned by Cerebrotech [68], instead, uses antennas transmitting different frequency radio waves for tissue bioimpedance detection, also this technology compares the bioimpedance in the two hemispheres exploiting the asymmetric nature of stroke events, but the VIPS device has two transmitters, located one on each side of the back of the head and a receiver in the forehead, therefore, the comparison of bioimpedance on each side is made for the whole hemisphere . The VITAL clinical trial [66] conducted on 252 patients has shown sensitivity of 93% and a specificity of 93% in detection of large vessel ischaemic stroke, evaluated as mean bioimpedance asymmetry. The ability to differentiate haemorrhage from ischaemia was not evaluated in this study [66].

Regarding BIS-based technology without phase shift there are two clinical studies [64,65]. The instrument used (impedimed SF7B) [64,65] is already on the market but is neither validated nor approved for the diagnosis of stroke. For this technology, the level is 7 according to the TRL scale. On the other hand, a large clinical study has been conducted on VIPS that has validated the effectiveness in the diagnosis of stroke. Cerebrotech [68] has also received premarket approval application from the FDA. The TRL level for this technology is 8 [60].

### 3.3. Microwave imaging system (MWI)

Microwave systems use antennas for microwaves (electromagnetic waves with a frequency band between 300 MHz and 300 GHz) transmission and for reflected waves reception. The scattering produced by the different dielectric properties [69] of biological tissues determines the reflection of the microwaves recorded by the sensors of the antennas. The dielectric properties (permittivity and conductivity) of the target, are functions of frequency [70]. The greater the differences between the dielectric properties of tissues, the greater the magnitude of the wave reflection. A frequency range of 1–4 GHz, for brain imaging show good trade-off for penetration and resolution [71], and the Federal Communications Commission approved a frequency range of 100KHz–10 GHz (specific absorption rate [SAR] = 1.6 W/kg per g of tissue) for MWI [71,72]. The data collected by the sensors are processed through different algorithms to return 2D and 3D images [73].

In biomedical field, MWI systems have been studied in breast cancer diagnosis [74].

The rationale for MWI use in stroke lesions comes from studies concerning the dielectric properties of brain tissue conducted on animals. Experimental data have shown that the electrical permittivity of haemorrhagic brain tissue is increased by 10–20% compared to healthy tissue, while in ischaemic tissue is reduced by 10–20% compared to healthy tissue [75]. The use of ultra-wideband (UWB) microwave [76] devices allows to reduce systems’ size, making them more compact and portable. In the present review, were found only three clinical studies concerning the use of microwave technology in stroke.

**Figure 5.**
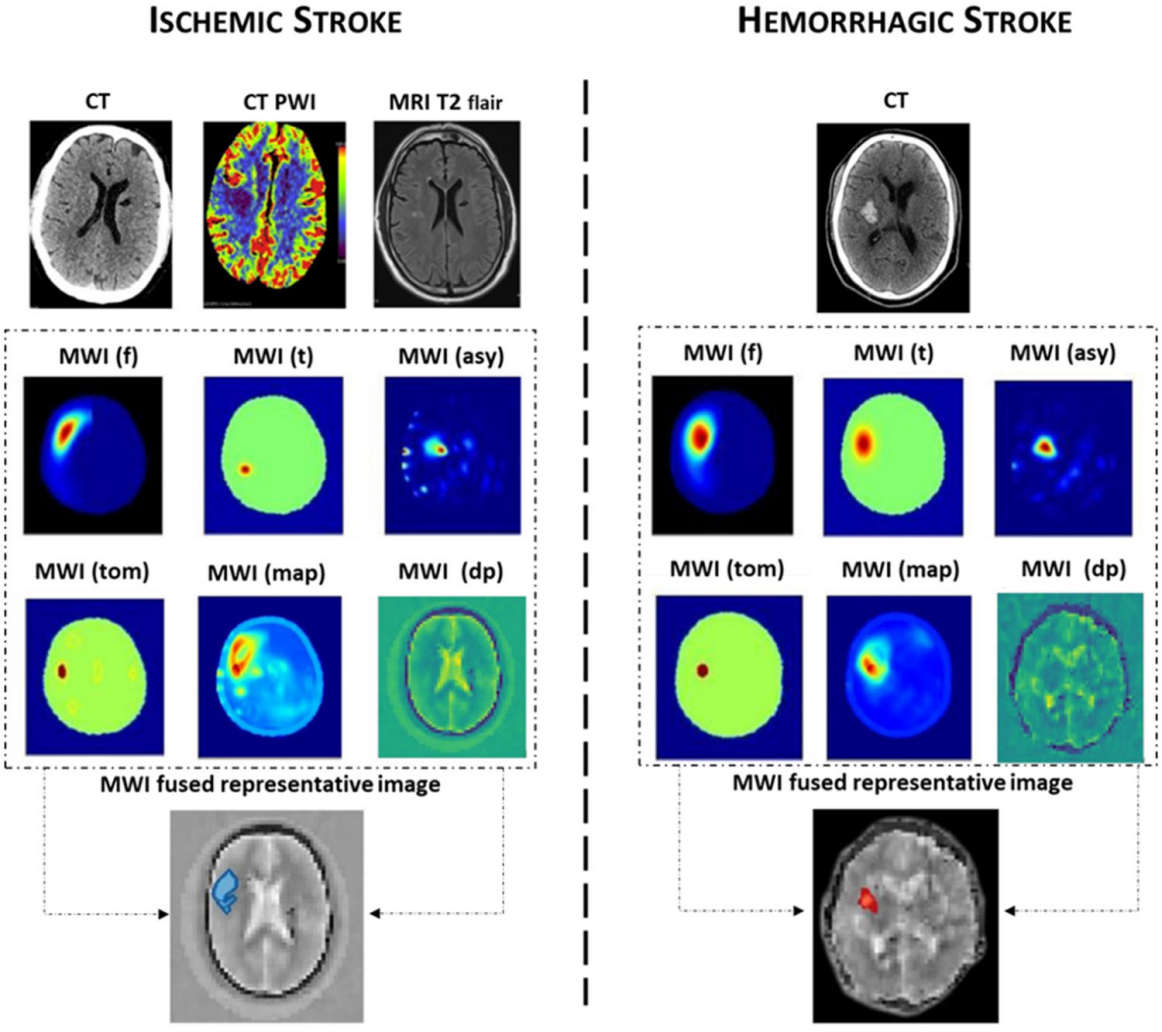
Microwave imaging (MWI). Left images: a patient with an ischaemic stroke right middle cerebral artery distribution. CT 3 h after onset. The plain CT shows no acute abnormality; however, the CT PWI (perfusion weight imaging) shows decreased blood flow in right middle lobe and MRI T2 FLAIR sequence shows small area of diffusion restriction at 24 h after symptom onset, and after almost complete resolution of symptoms. MWI (dp) dielectric permittivity map from an electromagnetic scan performed at 21 h after symptom onset. Right images: a patient with a haemorrhagic stroke, right basal ganglia. CT performed 2 h after onset, showing acute haemorrhage. Dielectric permittivity map from an electromagnetic scan performed 7 h after symptom onset. For both examples (ischaemic and hemorragic stroke) are showed examples of microwave imaging (MWI) processing algorithm using radar-based frequency domain [(MWI (f)] and time domain [(MWI (t)] signal analysis, signal asymmetry [(MWI (asy)], tomography [(MWI (tom)] and direct mapping [(MWI (map)]. The algorithm outputs are fused into a representative image for localization. An artificial intelligence algorithm classifies haemorrhagic stroke with a red colour code, while ischaemic stroke is colour-coded as blue, and this is overlaid on a greyscale permittivity map. (modified under the terms and conditions of the Creative Commons Attribution (CC BY) licence from Cook et al. [77]).

Cook et al. [77] reported two cases: one patient with ischaemic stroke and one with haemorrhagic stroke. Both were evaluated by CT, MRI, and MWI In the first case (ischaemic stroke) MWI was performed at 21 h from onset showing a right subcortical ischaemic lesion corresponding to the localization of the lesion found with MRI FLAIR performed at 24 h. In the second case (haemorrhagic stroke), the MWI conducted at 7 h from symptom onset showed a haemorrhagic lesion already evident at CT performed at 2 h. The device was able to recognize the lesions and differentiate its ischaemic or haemorrhagic nature (Figure 5) [77].

Persson et al. [78] investigated the diagnostic accuracy of two different brain diagnostic microwave devices in the differential diagnosis between ischaemic and haemorrhagic stroke and healthy patients. In the first device, the antennas were mounted on a bicycle helmet, in the second one on a custom system. Two clinical trials were conducted, the first on 20 patients studied in a time window of 7–132 h after stroke onset, 9 patients with intracerebral haemorrhage (ICH) and 11 with ischaemic stroke (IS). The second study enrolled 25 patients 7–25 h after onset, 10 patients with ICH and 15 with IS. In addition, a group of 65 healthy subjects was evaluated. The results showed that MWI was superior in differentiating ICH from healthy condition than differentiating ICH from ischaemia. The cause of low accuracy in the differentials between ICH and IS may be related to the significant oedema that develop a few hours after stroke onset [78].

Feasibility of using a magnetic contrast agent to differentiate ischaemic stroke from haemorrhage was evaluated by Hudson et al. [79]. Ferromagnetic nanoparticles were injected into animals and humans. In mice, MWI was able to evaluate a reduction of contrast diffusion in the side of the brain affected by ischaemia. In humans, only the ability to detect ferromagnetic contrast was evaluated. According to the study, the absence of contrast suggests an ischaemic event, instead its accumulation suggests an haemorrhagic event [79].

MWI is a relatively young technique that only in recent years had application in breast cancer although it is not yet used in the clinical setting. In stroke, there are only three small clinical trials and the first one was performed in 2014 [78]. Most papers on MWI still focus on an in lab evaluation using phantoms or animals [80–82]. The TRL, therefore, for this technology is 6–7 [60].

### 3.4. Diagnostic accuracy

Except for VIPS, no large sample size clinical trials have been conducted on NIRS, MWI or other BIS.

Regarding NIRS, the main points that influence the accuracy are the type of device (quantitative or qualitative NIRS) and the spatial coverage of the sensors. Q-NIRS is able to record absolute values of cerebral oxygen concentration and then to compare them with the same subject over time or with other subjects [55]. Moreover, Q-NIRS is not affected by changes in oxygenation of the skin and scalp, unlike qualitative NIRS [83,84]. About the spatial coverage of the sensors, studies show an increase in diagnostic accuracy and resolution proportional to the number of sensors [56,58,59].

The minimum size of the ischaemic lesion detectable by NIRS is not yet determined and despite a study reported that NIRS does not detect haematomas smaller than 3.5 mL in volume [53], the spatial resolution is determined by the probe density with a maximum spatial resolution around 10 mm [53,85,86]. The depth of detection of NIRS (max: 3.5 cm from the skin) is the major limitation to its use in stroke, indeed it is unsuitable in detecting deep lesions [48].

For BIS, the detection of the ictal lesion is defined in terms of bioimpedance asymmetry between cerebral hemispheres, thus a correct positioning of the electrodes on the head is essential [64]. Another important issue is the ictal lesion localization, since central or deep lesions, could go undetected by this technique. The reason is that, a central lesion may not determine an asymmetry between the hemispheres, instead in the case of deep lesion, it has been demonstrated that the deep parts of the brain contribute less to the value of the bioimpedance detected by the electrodes [64]. However, in VITAL trial, VIPS showed a global sensitivity and specificity of 93% in stroke detections [66].

For MWI technique, scattering data to images transformation algorithms are a crucial factor for the diagnostic accuracy. Most of MWI studies focus on the development of such algorithms, which can be classified into three groups: holography [87], tomography [88] and radar-based [89].

Another important factor is the interface between the antennas and the skin which can lead to microwave scattering [90]. Also for MWI the spatial resolution is strictly dependent on the number of sensors [91] even if the minimum detectable lesion size has not been evaluated in clinical studies conducted so far. One point in favour of MWI technique is that it allows the assessment of both superficial and deep lesions, thanks to the high penetration feature of microwaves.

### 3.5. Ability to differentiate between ischaemia and haemorrhage

NIRS is able to differentiate ischaemic lesions from haemorrhagic lesions, MWI showed good accuracy in differentiating between haemorrhage and healthy subjects, but the ability in discerning between ischaemia and haemorrhage was evaluated in only two patients. Regarding VIPS capacity in ischaemic from haemorrhagic stroke discrimination was not evaluated. In the single ICH patient evaluated, electric waves bioimpedance system was not able to discriminate the nature of stroke lesions [64].

In NIRS, ischaemia as assessed in different studies causes a reduction in blood flow detected as a reduction in O2Hb and a concomitant increase in HHb (given by tissue metabolism consuming the remaining oxygen) [55]. In a haemorrhagic lesion an increase in both HbO2 and HHb is detected by virtue of an increase in the total blood quota for extravasation [54].

Electrical permittivity analysed through MWI technique shows that haemorrhagic tissues can be differentiated from ischaemic ones, and, therefore, seems MWI to be able to discern the two different types of stroke [75]. However, cerebral oedema following ischaemic stroke can induce an increase in electrical permittivity, reducing microwave system diagnostic specificity [78]. MWI showed a ROC AUC in differentiating intracerebral haemorrhage from ischaemic stroke of 0.85. By increasing the sensitivity at 99.9% to detect haemorrhagic lesions, the proportion of ischaemic lesions identified was approximately 30%, whereas at 90% sensitivity 65% of ischaemic lesion was differentiated [78]. By using ferromagnetic contrast could improve the differential diagnosis between ischaemic and haemorrhagic lesions [79].

### 3.6. Time from stroke onset

VITAL study on VIPS evaluated patients before CT imaging, in the firsts hours from stroke onset [66]. Other BIS technique studies were evaluated only at 24 h from stroke onset [64,65]. NIRS has been shown to be able to detect ischaemic stroke even <3.5 h from onset [54]. MWI studies have been conducted using it not earlier than 7 h from onset [78].

### 3.7. Portability

The possibility to use these systems inside ambulances largely depends on the device size and energy needed. Compared to classic imaging techniques like CT and MRI, even in their portable format, neurophysiological techniques like NIRS, BIS and MWI are globally more portable and have a lower energy consumption. Several small portable NIRS systems are on the market including the FDA approved Model 1000 Infrascanner in subdural haematomas [53] as well some prototypes of portable MWI for stroke detection have been studied [82,91] with similar results in terms of accuracy to their larger counterparts. VIPS is a low size, portable helmet [66]. BIS, impedimed SF7B is a low size device associated with a simple EEG cap [64,65].

## 4. Discussion

The aim of this review was to evaluate the NIRS, BIS and MWI use as alternative methods to CT in the differential diagnosis of acute ischaemic and haemorrhagic strokes in order to safely begin thrombolytic infusion even before arrival at the hospital. For each of the three technologies, Table 2 summarizes the main findings in terms of: stroke *vs.* healthy and ischaemic stroke *vs.* haemorrhagic events diagnostic ability, spatial resolution, depth of penetration, time from stroke onset resolution, TRL (Table 3).

**Table 2.** NIRS, BIS, and MWI main findings for stroke diagnosis.

	Description	Stroke vs. healthy diagnosis	Ischaemia vs. haemorrhage diagnosis	Spatial resolution	Depth of penetration	Time from stroke onset resolution	TRL
NIRS	Detect Hb absorption of photons	YES	YES	10 mm	3.5 cm	Evaluate*d* > 3.5 h	7
BIS/VIPS	Analyze bioimpedance of tissues with radio waves	YES	NA	NA	Also deep lesions	Early (not described a mean onset time)	8
MWI	Analyze of scattering of microwaves	YES	YES	NA	Also deep lesions	Evaluated >7	6–7

TRL: technology readiness level

**Table 3.** Technology readiness levels description.

TRLs	Description
TRL 1	Basic principles are observed and reported
TRL 2	Technology concept and/or application formulated
TRL 3	Critical function, proof of concept established
TRL 4	Laboratory testing of prototype component or process
TRL 5	Laboratory testing of integrated system
TRL 6	Prototype system verified
TRL 7	Integrated pilot system demonstrated
TRL 8	System complete and qualified
TRL 9	System proved in operational environment

Technology readiness levels (TRLs).

Delay in the management of endovascular therapy is associated with worse outcome in terms of mortality and long-term disability. Moreover, the earlier the revascularization treatment is performed, the better the disability-free survival [92]. Healthcare costs are also closely associated with the timing of therapeutic intervention. Results show that a delay in endovascular therapy is associated with a reduction in Quality Adjusted Life Years (QALY) and economic value of care [93,94].

In evaluated studies, all techniques appear to be promising in stroke detection and ischaemic and haemorrhagic differential. However, there are many main limitations in the interpretation of the results related to the very limited number of studies that have been performed.

The sensitivity of MWI and NIRS seems to largely depend on the numbers of sensors and the spatial coverage they provide. Moreover, both MWI and Q-NIRS with multichannel system seems to be able to detect stroke lesion and distinguish haemorrhage from ischaemia. However, stroke lesions localization is a critical point for NIRS, in that it is able to detect only superficial strokes.

BIS clinical studies number is inadequate for assessing the capability of this technique in stroke detection and in ICH from IS differential diagnosis. With this technology, VIPS, is the only one with a large clinical trial that has shown high specificity and sensitivity in stroke detection. Moreover, it is the most ready technology between reviewed ones with a premarket approval application from FDA. However, the ability to differentiate haemorrhagic from ischaemic lesions has not yet been evaluated.

MWI diagnostic accuracy is not affected by stroke depth, but its ability to discern ICH from IS could be reduced in large oedema conditions that often occurs in large ischaemic stroke, however, using ferromagnetic contrast could lead to improve diagnostic performance in these cases. In addition, since the main aim of these technologies should be to exclude intracranial haemorrhage, in order to be able to start the thrombolysis, as happens with standard intrahospital CT scan, is better to have a high sensitivity for haemorrhage diagnosis even at the cost of low specificity. The rationale of this approach is to increase the number of patients eligible for thrombolysis according to timing criteria, in a safe way avoiding using thrombolysis in patients with haemorrhage lesions. With this strategy, in cases where an ischaemic stroke patient results as a false positive for haemorrhage, to MWI technique, due to the high degree of oedema, the thrombolysis can be initiated only in the hospital after ruling out haemorrhage with CT scan. Whit this, two steps diagnostic scheme, the total time to treatment for stroke patients population will sensibly decrease, improving the outcome in terms of disability and mortality, without wasting time for patients resulting false positive for haemorrhage at MWI, since the MWI analysis would be done during ambulance transport.

Techniques runtime are of milliseconds for MWI [95] NIRS [96] with an application time of only a few minutes. The timings for VIPS and BIS are not known. The biggest delay could be from fitting the helmet, but it would still be minutes.

Finally, unlike standard imaging techniques, neurophysiological techniques could allow continuous monitoring of patients, allowing for example intraoperative monitoring for the detection of stroke during endovascular or cardiac surgery, in addition to the timely intrahospital diagnosis of a restroke in patient hospitalized in stroke unit, even during night or in case of wake-up stroke in which is more difficult to identify alarming clinical symptoms of a restroke.

## 5. Conclusions

The present review shows how neurophysiology techniques could have a potential high impact in reducing the time to treatment in stroke patients. However, the small sample size and total number of clinical trials performed do not let to provide a high grade of evidence for these techniques, and, therefore, further studies are needed. The possible impact of these techniques on time-to-treatment time reduction could be up to 2 h, which means up to 240 million of neurons, 1660 billion of synapses, 1428 myelinated fibres saved and 7.2 years of accelerated ageing spared [6], and with an OR of 0.96 for mortality and symptomatic intracranial haemorrhage and 1.04 for ambulation independent at discharge every 15 min spared in time to treatment [8].

Since none of the proposed techniques is ready for clinical practice, in order to be used in future in clinical practice further and larger clinical trial are needed exploring the diagnostic performance (both stroke *vs.* healthy and ischaemia vs. haemorrhage), the spatial resolution, depth of penetration, testing patients in larger timespan from stroke onset with a special focus for the time window useful for thrombolysis (first 4.5 h).

## Data Availability

Data sharing is not applicable to this article as no new data were created or analysed in this study.
